# Compositional Properties and Colorimetric Characterization of Calcined Clays from the Central-West Region of Paraná and Their Application as Sustainable Pigment in Paints

**DOI:** 10.1021/acsomega.5c07843

**Published:** 2025-11-03

**Authors:** Anne Raquel Sotiles, Patrícia Appelt, Ricardo Schneider, Fauze Jacó Anaissi, Rafael Marangoni

**Affiliations:** † Department of Chemistry, 307046 Midwestern State University (UNICENTRO), Campus CEDETEG, Alameda Élio Antonio Dalla Vecchia, 85040-167 Guarapuava, Paraná, Brazil; ‡ Department of Chemistry, Federal Technological University of Paraná (UTFPR), Cristo Rei, 19, 85902-490 Toledo, Paraná, Brazil

## Abstract

This study investigated two clays from the Guarapuava region, Paraná, Brazil, which were calcined at different temperatures. Their compositional and structural properties were evaluated using diverse characterization techniques, and their colorimetric analysis was performed using the CIELab* system. Significant changes in the structural and granulometric properties of the clays were observed, as well as colorimetric changes in the samples that occurred due to calcination at temperatures of 200, 400, 600, 800, and 1000 °C. The clays were tested as pigments in colorless and white acrylic paints and applied to plaster blocks. Among the two clay samples, one (designated GC) exhibited a high quartz content and showed no significant structural changes even after calcination at 1000 °C, as confirmed by XRD analysis. In contrast, the sample designated BC, which presented the highest iron content via EDXRF analysis, demonstrated the most significant structural alterations and phase changes in XRD, TGA, and particle size tests. Furthermore, BC yielded more pronounced color variations due to changes in the phases of its iron compounds, indicating its greater potential for use as a pigment.

## Graphical abstract

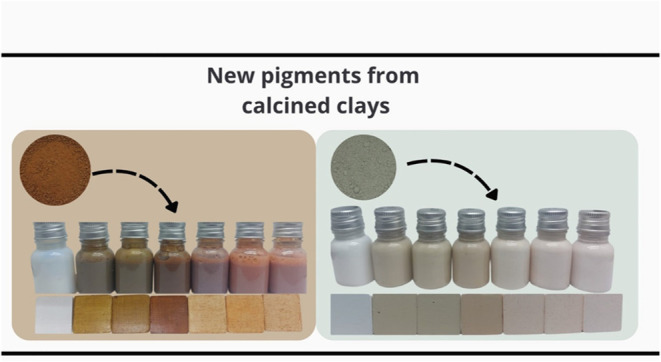

## 1 Introduction

Calcined clays can present different properties when compared to natural clay, such as changes in porosity, surface area, mineralogical composition, chemical reactivity, greater resistance, varied color, and high adsorption capacity for chemical substances. In addition, they are an important essential ecological raw material in the production of construction materials^1–3^

Among these characteristics, color plays a prominent role in clay studies. The color property of clays varies depending on their composition and is generally influenced by the iron content and compounds present. During the calcination process, the color can be modified, which is directly related to two main factors: the oxidation state of the iron oxide present in the raw clay, the phases in which iron is found in clay, and which iron-containing compounds are formed during the calcination process.^4–6^

The iron found in clay samples can influence several aspects, whether present in forms such as hematite, magnetite, and goethite, among others, or the degree of hydration of these compounds. These different phases enable the occurrence of redox reactions, especially at higher temperatures (oxidation of Fe^2+^ to Fe^3+^) or in transformations from one phase to another through dehydration or dehydroxylation, which can expand the areas of application of these compounds.^4,5,7,8^

The applicability of calcined clays has been known since ancient times, such as in the manufacture of clay bricks or ceramic utensils. Recently, they have been utilized in various areas, including the formulation of cosmetics, enamels, and water treatment as contaminant adsorbents, as well as primarily as supplementary cementitious materials (SCMs). The latter is becoming increasingly attractive and ecological for the cement industry, as the use of pozzolanic and/or latent hydraulic cement instead of Portland cement clinker represents a reduction in CO_2_ emissions in the cement industry.^3,9^

Metakaolinite, obtained from the calcination of kaolinite, is one of the SCMs added to cement. Also, calcined clays in association with a source of lime (CaO or Ca­(OH)_2_) have been used in the production of lime-pozzolan cement, being able to improve the strength and durability of concrete or mortar.^3,10^

Brazil, with its vast territory and geological diversity, is one of the world’s largest producers of clays, including kaolin, bentonite, and illite. For instance, Brazil’s kaolin reserves are estimated at 4144 Mt, with 90% located in the Amazon region.^11^ This mineral wealth drives research into its characteristics and applications across various sectors, including ceramics, petroleum, cosmetics, pharmaceuticals, and environmental sciences, positioning the country as a hub for technological and scientific development in this field.^12–14^

Due to the large abundance and heterogeneity of clay samples found in the country, it is crucial to study locations that contain clays of interest for future applications. In this work, we conducted a study involving two clays from the Guarapuava region, located in the central-western state of Paraná (Brazil), known for its richness in these materials, to analyze the influence of temperature on the structure and, consequently, the color of both samples. The samples were then dispersed in paints to assess the viability of their application as natural pigments, since they are sustainable, low-cost, and widely available materials that can produce different colors simply by subjecting the samples to heat treatment.

## 2 Experimental Section

### 2.1 Calcination of Clays

The two clays were obtained in the region of Guarapuava, Paraná, Brazil (25°23′36.0″S 51°27′57.3″W). The clays were collected and underwent only the steps of drying in an oven at 100 °C for 48 h and then grinding, using a mortar and pestle. There was no additional treatment. The clays were named based on their initial colors: gray (GC100) and brown (BC100). They were then calcined in porcelain crucibles in a muffle furnace. The samples were subjected to a calcination process with a heating rate of 5 °C/min. The samples were held at the calcination temperature for 2 h. The temperatures used for the process were 200, 400, 600, 800, and 1000 °C. The samples were named based on the corresponding clay code and calcination temperature after the calcination process. The loss on ignition (LOI) was determined by eq 1.

1 $$\mathrm{LOI}=\frac{Z-Z^{*}}{Z}\times 100$$

Where *Z* is the initial mass of the sample and Z* corresponds to the mass after heating at a temperature.^15,16^

### 2.2 Dispersion in Paint

The GC and BC clays were applied as pigments at a ratio of 10% before and after calcination in water-based acrylic paint in white and colorless colors. The pigmented paints obtained were used to square blocks measuring 25 × 25 × 15 mm (*W* × *L* × *H*), previously prepared with plaster. The application was done with a brush, and the paint was dried at room temperature for 24 h.

### 2.3 Clay Powder Characterization

The samples were characterized by different techniques. X-ray diffraction was analyzed in SmartLab SE Rigaku 3 kW equipment, operating at 40 kV and 30 mA. The scan was performed from 4 to 70° of 2θ, with a speed of 10°/min and a sampling field of 0.02°.

For the Zeta potential analysis, the sample was dispersed in water (0.1% m/v), and the analyses were performed in Zetasizer Malvern Nano series equipment model Nano-ZS90, using 3 measurements of 20 runs each and operating at 20 °C.

Characterization by energy dispersive X-ray fluorescence (EDXRF) was performed using a plastic sample holder measuring a diameter (collimator) of 3 mm, which was inserted into the carousel in a Shimadzu model EDX-7000 equipment with an Rh tube operating at air atmosphere and 50 kV with an Al–U state detector and 15 kV for high-resolution Na–Sc and S–K detectors, and an integration time of 100 s.

SEM analyses were carried out at the National Nanotechnology Laboratory (LNNano) and the National Center for Research in Energy and Materials (CNPEM) using a Thermo Fisher Scientific Inspect F50 microscope. The samples were covered with a carbon layer, and the images were acquired using a 5 kV voltage and a secondary electron detector.

To determine the particle diameter of the particles, analyses were performed using Static Light Scattering (SLS) on a Horiba LA-960 instrument, with water as the dispersion medium.

Colorimetric analyses of powders and paints were performed in a portable colorimeter (NR60CP–3NH). The method used to evaluate color is CIEL*a*b*, which defines colors in three dimensions: L* (brightness), a* (red-green coordinate), and b* (yellow-blue coordinate). In this system, it is possible to compare the difference between two colors, known as Δ*E*, and calculated according to eq 2. Generally, the human eye cannot differentiate between colors with a Δ*E* less than 2.0 units.^17^

2 $$\Delta E_{\mathrm{ab}}^{*}=\sqrt{{\left(\Delta L^{*}\right)}^{2}+{\left(\Delta a^{*}\right)}^{2}+{\left(\Delta b^{*}\right)}^{2}}$$

Where ΔL* is the luminosity variation, Δa* is the variation of parameter a* (green-red), and Δb* is the variation of parameter b* (blue-yellow).^17,18^

## 3 Results and Discussion

### 3.1 Clay Powder Characterization

In the characterization by X-ray diffraction (Figure 1), the BC and GC clays showed similarities in the results, with both clays showing a mixture of phases, with a predominance of signals referring to quartz, whose crystalline phase corresponds to SiO_2_ (Code 16631). Other studies also report clays abundant in quartz.^19,20^ In addition to quartz, sample BC100 (Figure 1A) exhibited characteristic signs of the presence of kaolinite (Code 68698), with a predominance of the main signals close to 12° and 25° of 2θ referring to the [001] and [002] planes, respectively, with basal distance of 7.35 Å, in addition to the signal at approximately 20° of 2θ, referring to the [020] plane.^21,22^ Signals attributed to the goethite phase (FeOOH) (Code 245057) are observed in the regions of 22°, 28°, 38°, 55°, and 64° of 2θ, consistent with the literature that describes that quartz and kaolinite are frequently associated with iron oxides and metallic ores.^23^

**Figure 1:**
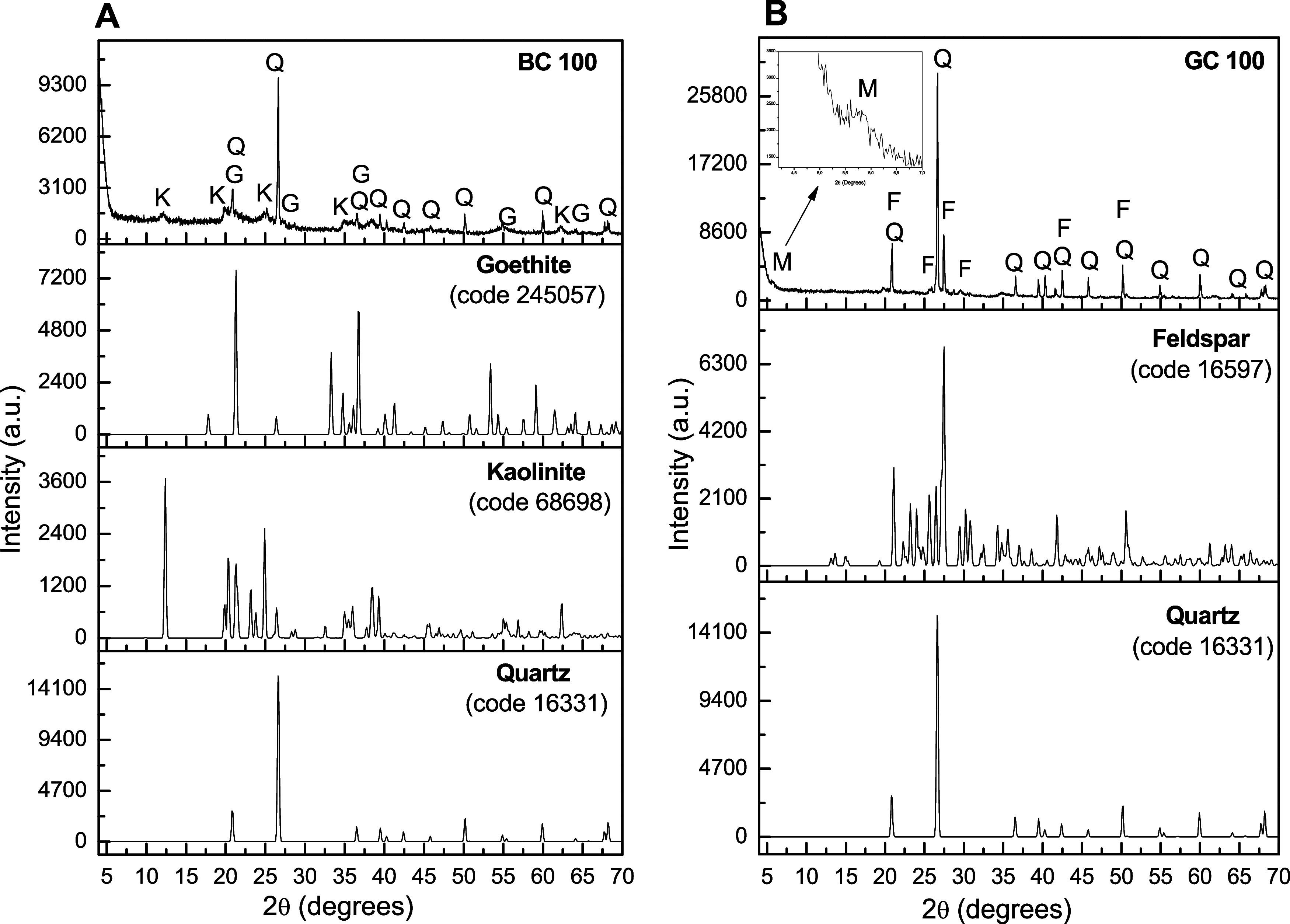
X-ray diffraction patterns of (A) BC clay samples compared with quartz (Q), goethite (G), and kaolinite (K) standards and (B) GC clay compared with quartz and feldspar (F) standards and montmorillonite (M).

The X-ray diffractogram of sample GC100, visualized in Figure 1B, signals attributed to the presence of feldspar (Code 16597) and a sign of smectite-type clay, with a characteristic peak of the montmorillonite phase in the region of 5° of 2θ, referring to the basal plane 001 of montmorillonite (Code 9002779), with a basal distance of 15.21 Å. The result is similar to a clay reported in the same region, but without feldspar.^24^

The compositions obtained by EDXRF are consistent with the XRD analyses, indicating a high silicon content in the samples (Table 1). The BC samples had a higher aluminum content than the GC samples due to the presence of kaolinite. The high iron content in the BC samples can be attributed either to the goethite phase initially identified in XRD, or it may be in the kaolinite structure since iron can replace aluminum isomorphically.^25^ Furthermore, kaolinite commonly presents titanium contamination, which explains the presence of this element in BC samples. Other studies have already shown iron and titanium contamination in kaolinite.^26–28^

**Table 1:** Composition of Samples Obtained from EDXRF

sample	SiO_2_ (%)	Al_2_O_3_ (%)	K_2_O (%)	CaO (%)	Fe_2_O_3_ (%)	TiO_2_ (%)	other elements (%)
BC100	48.579	19.357	0.529	0.248	22.437	7.859	0.668
GC100	74.549	10.122	6.031	5.579	2.272	0.779	0.991

In addition to silicon, GC clay samples showed high calcium and potassium levels. This can be attributed to the presence of calcium montmorillonite (Ca-montmorillonite), which is very common,^29^ and feldspars, commonly potassic feldspars (K-feldspars). Feldspars are light minerals often found in surface rocks and silty materials.^30^ The iron present may be replacing aluminum in octahedral sites or occur in oxide or hydroxide phases such as hematite and goethite, among others, even if it is not evident in the diffractograms of the GC samples due to the predominant signals from the other clay phases. The identified elements are also consistent with those previously reported for clay from the same region.^8,24^

It is known that in mixed mineral phases, the intensity of the signals in XRD depends on the physical properties of the materials and, therefore, can make it difficult to evaluate structural differences.^31^

Clay samples showed notable changes in the structure of the phases present after calcination at different temperatures (Figure 2). Only the modifications that occurred were highlighted in the diffractograms. After reaching a temperature of 600 °C, sample BC600 (Figure 2A) no longer exhibited the characteristic signs of kaolinite. This is since, generally, at temperatures above 400 °C, kaolinite dehydroxylation occurs, converting it into metakaolinite, which has a more amorphous characteristic, which was not observed in the XRD analysis, as the intensity of the quartz present in the sample stands out and even after heating at different temperatures the quartz remains.^5,32^ Some reported clay samples showed mullite formation at temperatures above 900 °C, but the mullite phase was not observed in sample BC1000.

**Figure 2:**
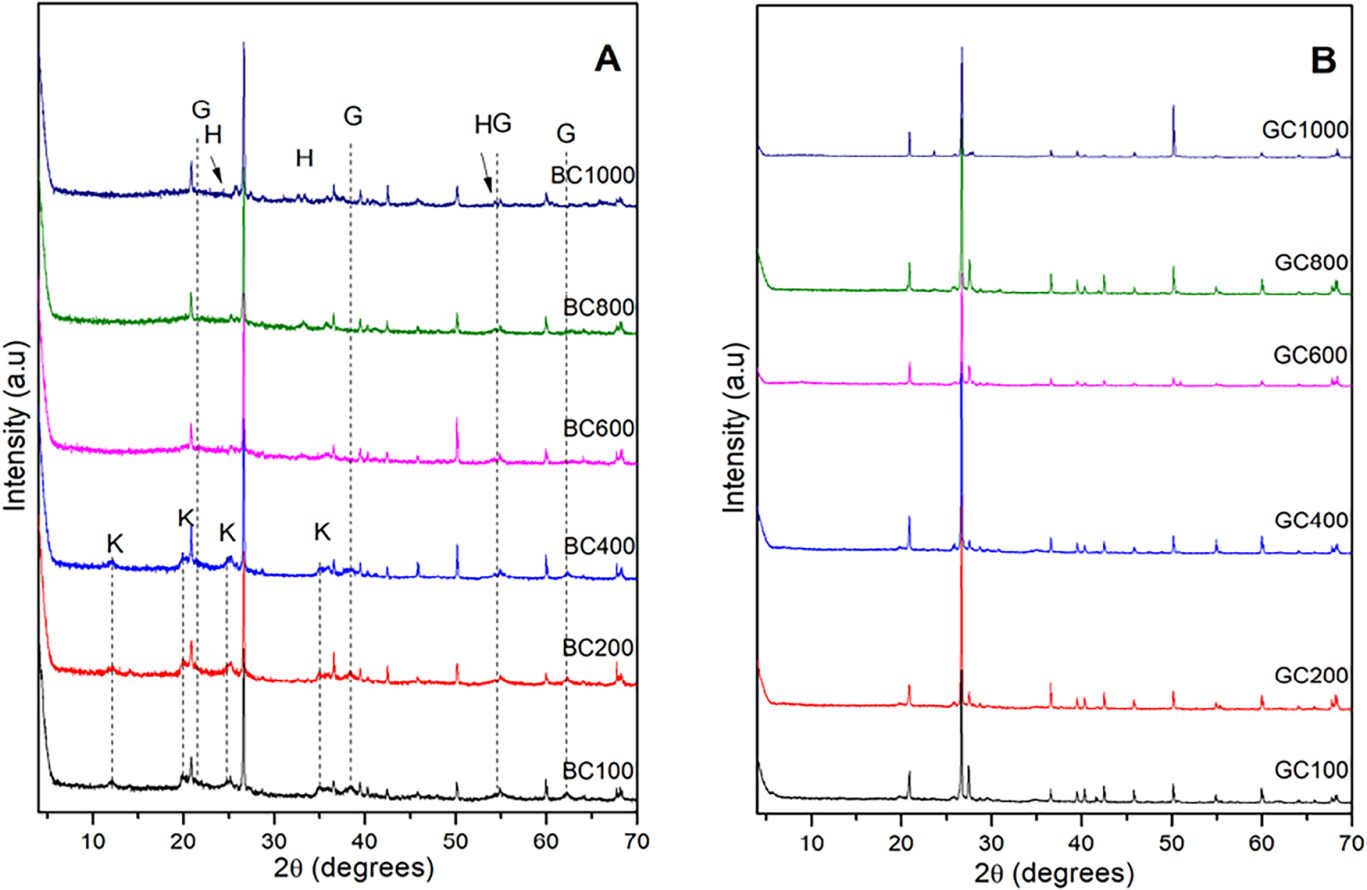
X-ray diffraction patterns of clay samples BC (A) and GC (B) after calcination at different temperatures. G-goethite, K- kaolinite, H- hematite.

The signals referring to goethite are observed until sample BC400 and are subsequently absent, replaced by new signals attributed to the hematite phase in the region of 24°, 32°, and 54° of 2θ (Code 2101167), formed by iron oxidation. This transformation is described in eq 3 and occurs at temperatures near 300 °C. However, the temperature is dependent on the availability of the element, whether the iron is free or bound, as in the case of isomorphic substitutions in kaolinite.^33,34^

3 $$2\mathrm{FeOOH}\to {\mathrm{Fe}}_{2}O_{3}+H_{2}O$$

The GC sample (Figure 2B) did not show significant changes; the signs attributed to the presence of feldspar persisted even in the GC1000 sample, being compatible with the results observed in other studies, which describe that decomposition of feldspar begins only above 1000 °C.^33^ In the GC sample, a reduction in the intensity of reflections attributed to quartz is observed, which may be related to the transformation of α-quartz into β-quartz, changes that occur at temperatures close to 573 °C and involve a change in symmetry, in addition to the fact that the increase in temperature causes contraction along the *c* axis in this second structure.^34–36^

Phase changes in minerals lead to various changes in physical and chemical properties. The phase transition from α-quartz to β-quartz is no different, and at high temperatures, the observed phase is already β-quartz. The materials present less variation in properties, while at values close to the α → β transition, nonlinear variations in several properties are observed due to the possibility of the two quartz phases coexisting.^35,37^

The loss of ignition (Table 2) presented during heating at different temperatures is possibly associated with dehydration and losses of organic matter present in the samples. Still, the results obtained at the highest temperatures of 800 and 1000 °C for the BC sample that contains kaolinite in the composition are close to those reported for a clay containing kaolin heated to 800 °C that presented an LOI of 12%^15^ and kaolinite calcined at 1200 °C that presented an LOI of 13.65%.^32^ The GC sample presented an LOI of up to 5.46%, even lower than those reported for different clays such as illitic clay (7.10%)^38^ or sodium bentonite (9.80%).^15^

**Table 2:** Loss of Ignition Data for Clay Samples at Different Temperatures of Calcination

sample	initial weight (g)	final weight (g)	loss on ignition (%)
BC100	-	-	-
BC200	50.0025	48.5843	2.8363
BC400	50.0169	47.9995	4.0334
BC600	50.0013	47.9365	4.1295
BC800	50.0089	43.0845	13.8463
BC1000	50.0076	42.9425	14.1281
GC100	-	-	-
GC200	50.0024	48.3819	3.2408
GC400	50.0045	48.3782	3.2523
GC600	50.0010	47.7849	4.4321
GC800	50.0093	47.3791	5.2594
GC1000	50.0037	47.2757	5.4556

The thermogravimetric curves show that the clay samples (Figure 3) present initial mass loss up to a temperature of 100 °C, which is associated with dehydration. Even though the analyzed samples BC100 and GC100 had been previously dried, they reabsorbed moisture, as also occurred in the smectite and vermiculite clay samples previously dried and subsequently thermally evaluated.^34^ The BC and GC samples presented mass loss values related to dehydration of approximately 3.38% and 1.78%, respectively.

**Figure 3:**
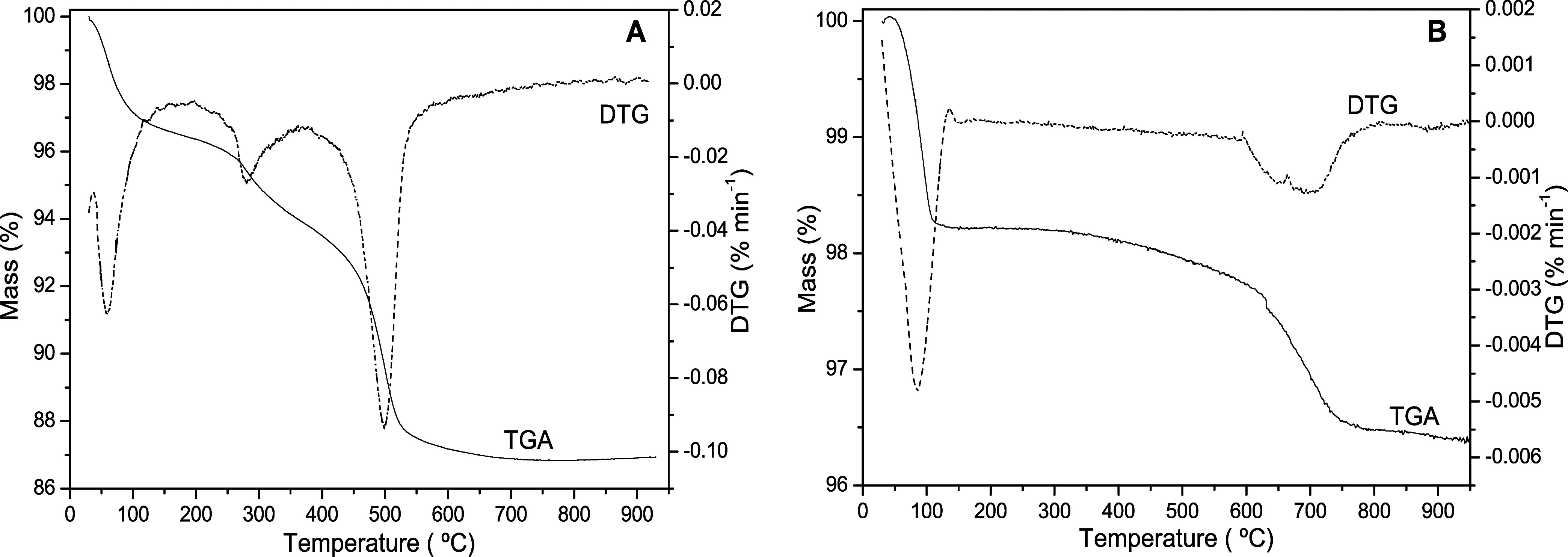
TGA/DTG curves of clay samples BC (3A) and GC (3B).

The BC sample (Figure 3A) showed a second mass loss event (approximately 2.39%) in the region of 200 to 350 °C and is related to the transformation of goethite into hematite.^39^ The third event, from 420 to 550 °C, corresponds to the dehydroxylation of kaolinite and its transformation into metakaolinite, as evidenced by the XRD analysis (Figure 2), where, up to the BC400 sample, the signal referring to kaolinite is observed. Still, from sample BC600, the signal is no longer observed, indicating the transformation into metakaolinite.^34^

The GC sample (Figure 3B) exhibited only one mass loss event, aside from dehydration. This event occurs within the range of 600 to 750 °C and primarily refers to the dehydroxylation of smectites, such as montmorillonite, which is present in sample.^34,40^ The GC sample appears not to have completed the decomposition process at the final temperature analyzed, agreeing with the X-ray diffractograms (Figure 2), in which it was evident that at a temperature close to 1000 °C, the decomposition of the feldspar phase began.

The SEM images of the GC samples are more compact and aggregated than the BC sample (Figures S1 and S2). Regarding heat treatment, it is observed that BC samples begin to disaggregate particles at a temperature of 400 °C (Figure S1c) into narrower platelets, possibly due to the conversion of kaolinite into metakaolinite^41^ since this mineral is present in BC clay. Except for the temperature of 1000 °C, at which the clay samples do not appear to have initiated the sintering process, and they do not exhibit significant changes in morphology due to the heat treatment at different temperatures.

In general, the EDS spectra (Figure S3) showed high silicon contents due to the presence of quartz and elements consistent with those observed in the EDXRF analysis. As in the case of the EDXRF analysis, the BC sample (Figure S3A) showed titanium contamination and higher levels of Al and Fe than the GC sample, while the CG sample showed Ca due to the presence of calcium montmorillonite (Figure S3B). The presence of carbon is justified by the covering of the material used to take the SEM images.

Similarly, no significant changes were observed in pH and Zeta potential between clay samples before and after heat treatments (Table 3). It is still unclear to what extent the Zeta potential is influenced by clay calcination.^42^ However, changes in potential and pH values may be related to phase changes evidenced by heating, with the different cations present, which, even in small proportions, can influence the surface properties of the clay.^43^

**Table 3:** Data on Zeta Potential, pH, Mean Size, and Surface Area of Particles of Clay Samples

sample	zeta potential (mV)	pH	mean size (nm)	surface area (cm^2^/cm^3^)
BC100	–19.30	7.54	45.928	3257.80
BC200	–18.40	7.50	52.398	3306.60
BC400	–18.77	7.57	151.915	2034.30
BC600	–17.43	7.75	295.787	1125.40
BC800	–20.63	7.70	284.067	1190.00
BC1000	–23.83	7.86	276.529	1140.00
GC100	–21.50	9.81	103.858	2792.60
GC200	–19.53	9.78	82.509	3104.30
GC400	–21.60	9.72	80.632	2360.90
GC600	–20.87	9.20	109.846	1029.80
GC800	–20.60	9.61	113.381	955.60
GC1000	–11.37	9.19	124.811	708.42

The samples showed a negative Zeta potential value, as expected for clays. The GC sample showed a more negative Zeta potential than the BC clay, probably due to the higher pH value in these samples.^42^ Sample GC1000 showed the most significant variation in Zeta potential when compared to other samples of the same clay calcined at different temperatures. When calcined at 1000 °C, the decomposition of the feldspar was evident (Figure 2B), in addition to the fact that the sample presented a sandier characteristic, and these values could be associated with the difficulty in dispersing the sample in water and the rapid sedimentation of the particles during the measurement of Zeta potential.

The average size of BC particles increased with increasing calcination temperature, up to a temperature of 600 °C, and showed a slight decrease at temperatures of 800 and 1000 °C, which may be related to the transformation of kaolinite into metakaolinite and later mullite.^32^ In the case of the GC sample, there was initially a reduction in the average particle size up to a temperature of 400 °C (80.632 nm) when compared to the initial sample (103.858 nm), and subsequently an increase in the average value up to a temperature of 1000 °C. Figure 4 shows the particle size distribution histograms of the clays, and it can be seen that in the case of the BC sample, there is a shift toward larger sizes, while in the case of the GC sample, no large shifts are observed. The individual histograms of the BC and CG samples at each heat treatment are shown in Figures S4 and S5.

**Figure 4:**
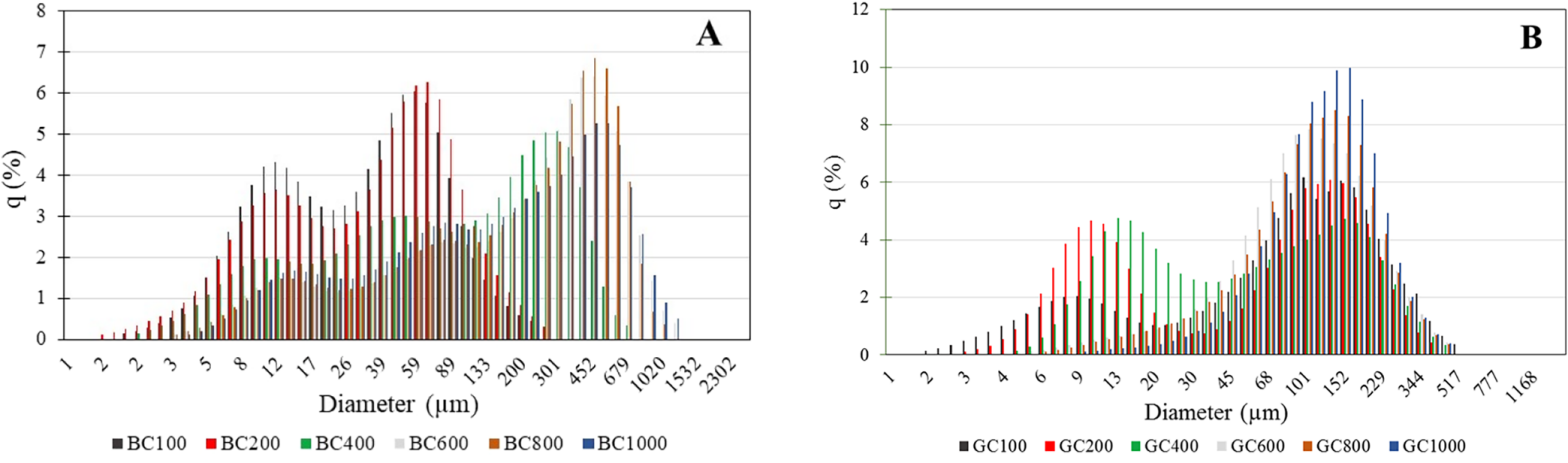
Histograms of particle size distribution of samples BC (4A) and GC (4B) at different temperatures.

Individually observed, the histograms of the BC sample present a distribution divided into two signals at temperatures of 100 and 200 °C, and when calcined above 400 °C, this distribution becomes divided into three signals. This is characteristic of clays, as during calcination, particles may also be agglomerated in addition to an increase in particle size, resulting in a new signal.^44^ In the GC sample, the initial particle size distribution is observed to have two peaks. After calcination at 600 °C, only one signal remains, which retains the same position as one of the previous signals, indicating that the average particle size has increased. The surface area of the two samples, BC and GC, decreased after heat treatment at temperatures above 200 °C, in line with previously reported results for different clays and minerals.^32,45,46^

### 3.2 Colorimetric Analysis and Dispersion in Paint

The data relating to the colorimetric measurements of the samples, together with the images obtained from the samples in powder form, are presented in Table 4. It can be observed that there was a change in color and luminosity as a function of the calcination temperature. This color change may be related to phase changes and the iron in the sample’s composition, which can alter its structure and color depending on the degree of hydration.^47^ Given that the luminosity parameter (L*) ranges from 0 (black) to 100 (white), the samples exhibited an increase in the L* parameter as the calcination temperature increased, with values ranging from 76.45 to 99.99 for the sample BC and 30.40 to 52.02 for the GC sample. In both cases, the sample calcined at 800 °C showed lower luminosity than obtained at 1000 °C.

**Table 4:**
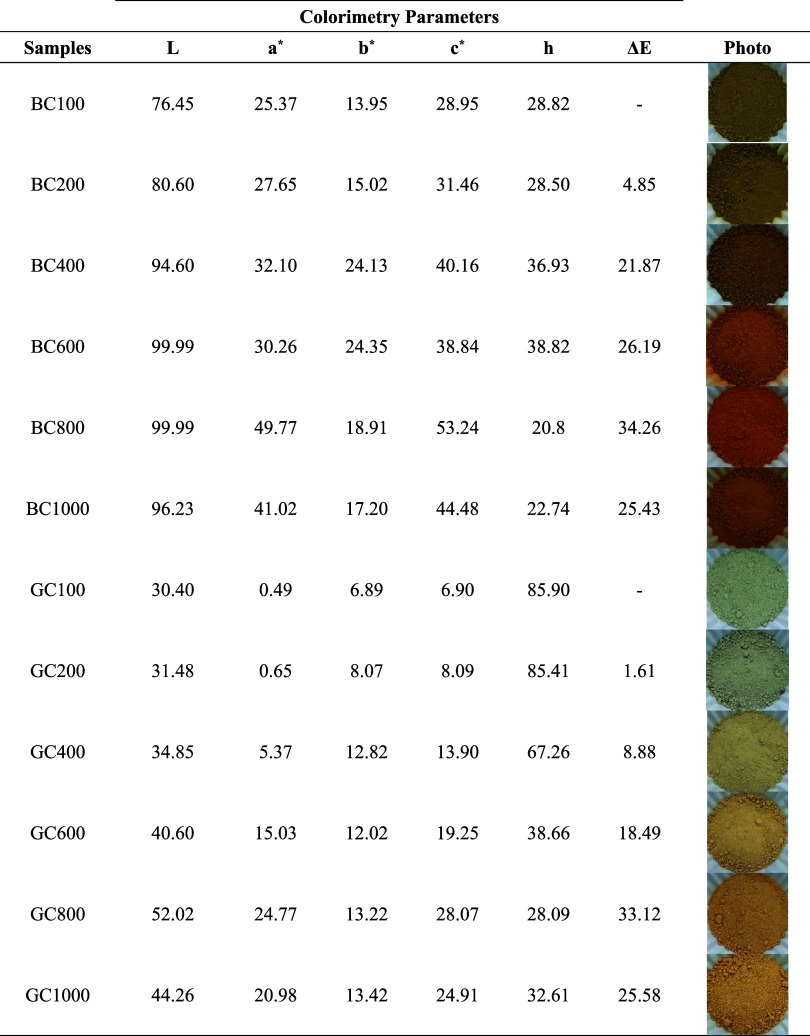
Colorimetric Parameters of GC and BC Clays Calcined at Different Temperatures and in Powder Form

To facilitate visualization of the color scale, a graph was plotted (Figure 5) relating the a* and b* parameters of the samples, according to the CIELAB system. The initial samples of BC and GC clays, which had a more brown and gray color, respectively, started to show a shift in the colorimetric result in the positive direction of the a* axis toward more reddish tones. There was also a shift in the positive direction of the b* axis, which is characterized by positive values tending toward yellow and negative values toward blue. This change to positive values in both axes occurred as the calcination temperature was increased, indicating that the samples became more orange in tone. Studies carried out with different clays have already reported that, after calcination, the clay presents an orange or reddish color due to the presence of iron-containing phases^4,7,8,48^ and, in EDXRF analyses, relatively high iron values were obtained (values close to or above 10% for GC and 50% for BC).

**Figure 5:**
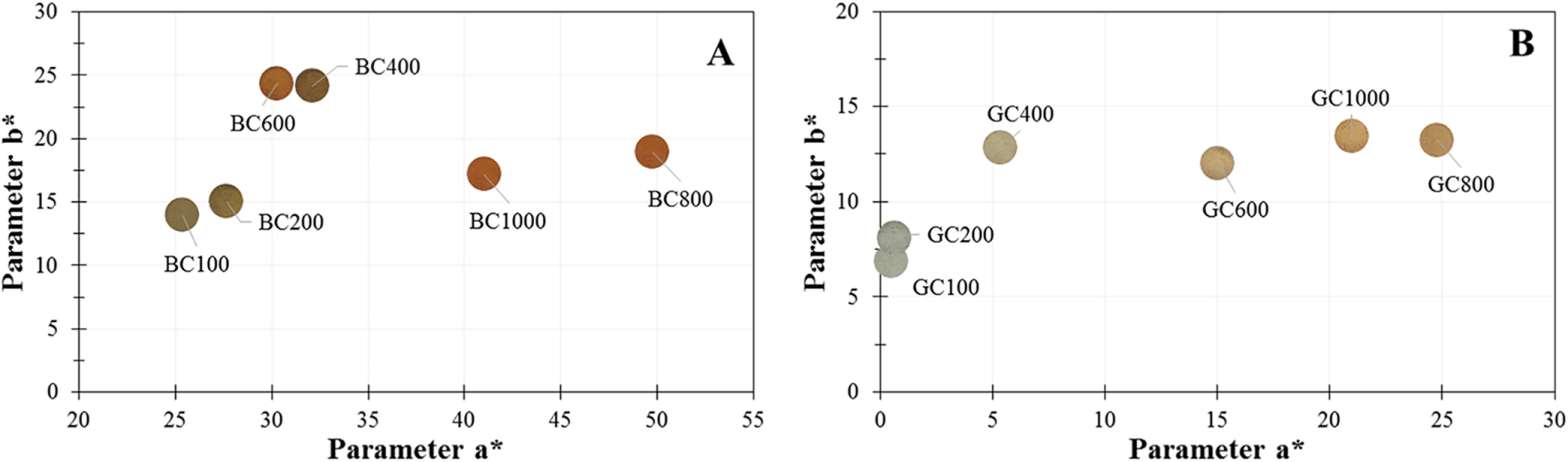
Relationship between the colorimetric parameters a* and b* of clays BC (A) and GC (B) at different heat treatment temperatures.

This color change can be seen in the images shown in Table 4. As well as the behavior of the L* parameter, the BC1000 and GC1000 samples also presented lower values of the a* parameter than the respective samples obtained at 800 °C. This may be associated with the fact that at a temperature of 1000 °C, the oxidation of Fe^2+^ to Fe^3+^ can occur, resulting in a darker, reddish-brown color (decrease in luminosity).^7,8^ Studies report that the hematite phase obtained, as confirmed by the X-ray diffractogram (Figure 2), which undergoes heat treatment at high temperatures, produces a darker color than those treated at lower temperatures.^49^

The color variation values (Δ*E*) were calculated using the respective initial samples BC100 and GC100 as a reference (Table 4), and it is possible to notice that the Δ*E* value also increases with increasing firing temperature and decreases in samples of 1000 °C. According to the visual color distinction classifications between samples, samples BC200 and GC200 presented an Δ*E* of 4.85 and 1.61, respectively, being considered a distinguishable color from the respective precursor samples BC100 and GC100. Sample GC400 presented a Δ*E* above 6, resulting in 8.88 being classified as a significant color distinction from the initial sample GC100. The other samples after calcination had Δ*E* values above 12, classified as a considerable color variation from the precursor samples.^50^

Due to the different colors obtained, the samples may be promising for application as sustainable pigments, and other clay samples with possible application as pigments have already been reported in the literature,^5,51–54^ but there are still few studies that have effectively applied and evaluated clays as pigments in paints, and when applied, the clays were not used directly, but rather in the form of hybrid pigments, incorporated into other compounds.^55–57^

The samples were then added at a proportion of 10% as pigments in colorless paint and white paint and applied to plaster blocks (Figure 6), which were again evaluated by colorimetric analysis. The parameters are depicted in Tables S1 and S2, as well as respective color images. The luminosity values of the pigmented paints were very close to each other but different from the respective samples in powder form (Table 4), as the luminosity of the paint prevails. As expected, the exact behavior of the samples in powder form was observed, where the colors of the samples showed an increase in the a* and b* parameters as a function of the heat treatment.

**Figure 6:**
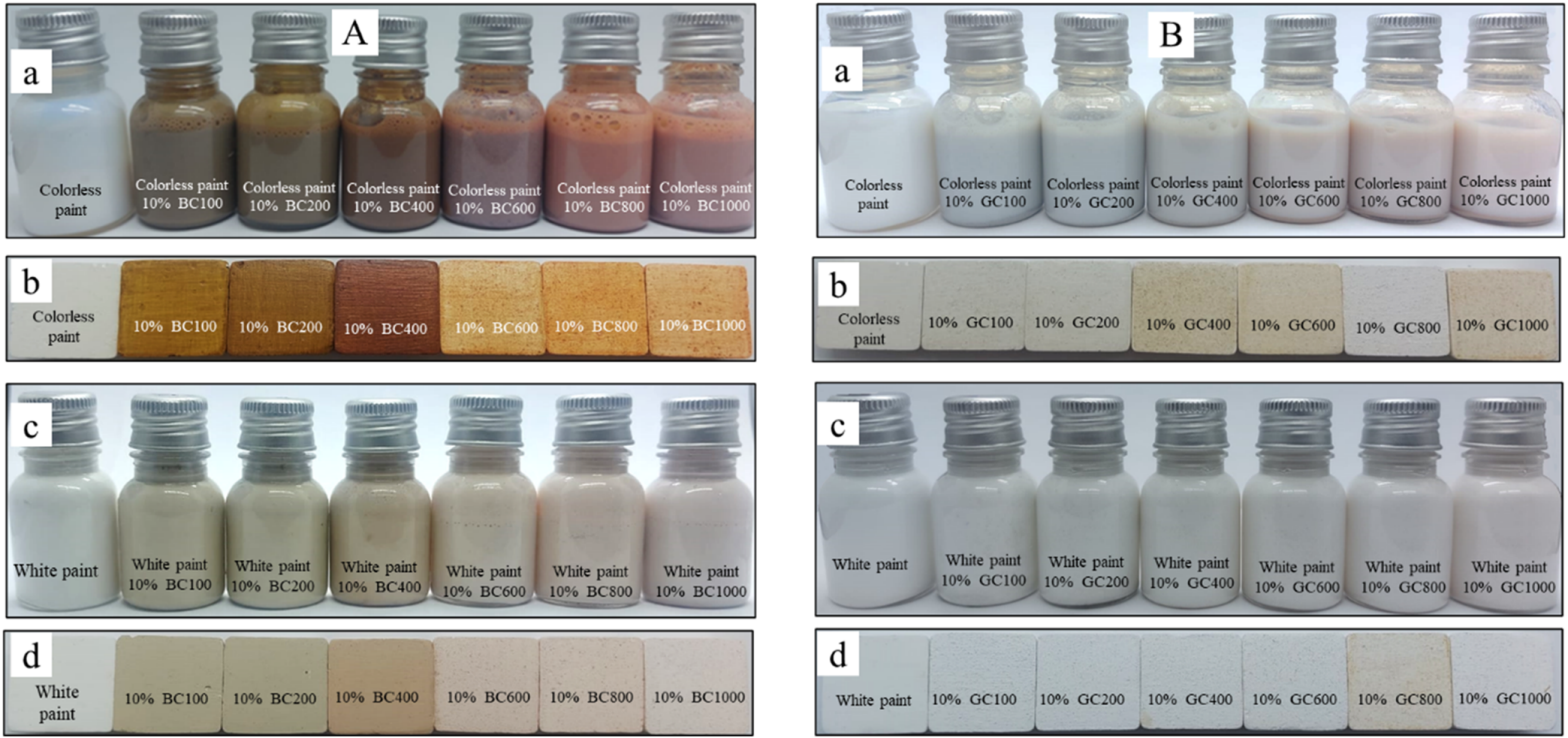
BC clays (A) and GC clays (B) were used as pigments in colorless paint (a) and applied to plaster blocks (b) and in white paint (c) and applied to plaster blocks (d).

In the case of the BC samples, the Δ*E* value calculated in relation to colorless paint or white paint was above 12 for all samples, indicating a large color discrepancy compared to the precursor samples (blocks painted only with the paint, without pigment).^50^ For the GC sample, the calculated ΔE values showed fluctuations. In general, the Δ*E* values were higher for colorless paint, as a color dilution effect can occur in white paint due to the presence of the white pigment. This is also visible by the chromaticity value (parameter c*), which represents the color tone and decreases when clays are applied to white paint. The same effect was observed in the shade of green Co/Zn oxide pigments used to white paint.^58^ However, among the clays and paints evaluated, BC clay was the one that presented the most significant possibility of obtaining colors and tones, showing that the composition of the sample, mainly the presence of iron and high content, and the transformations that occurred during the process of calcination, influence the color of the material.

## 4 Conclusion

The presented work demonstrates qualitatively that the composition of the two clays studied can influence material properties, such as Zeta potential and particle size, as well as colorimetric changes linked to structural alterations, both of which occur when the clays are calcined at different temperatures.

The XRD results show that the BC clay initially presented a mixture of quartz, kaolinite, and goethite phases. After calcination at different temperatures, the goethite was transformed into hematite, and the kaolinite was dehydroxylated and transformed into metakaolin. Although the amorphous halo of the metakaolin could not be observed due to the overlap of the quartz crystalline signals, the disappearance of the kaolinite signals was notable, confirming dehydroxylation. In the case of the GC clay, the sample composed of quartz, potassium feldspar, and calcium montmorillonite did not show significant changes in the diffractograms after calcination.

Parameters such as Zeta potential and pH of the clays did not change significantly with calcination. Particle size generally increased when the sample was heated to 600 °C and then decreased; possibly, in addition to the increase in size, there was agglomeration of the particles.

Regarding color changes, the sample with the highest iron content, BC clay, showed the most significant color variation with calcination, possibly due to iron phase transformations. However, both clays may be promising when applied as pigments in water-based acrylic paint, although GC clay better displays the color of the colorless paint. The use of clays as pigments in paints is promising because they are an abundant and widely available material, and because of the possibility of obtaining a variety of colors through calcination at different temperatures or even by mixing clays with different compositions. It also reduces waste, costs, and contaminants that can be generated in the production of synthetic pigments.

## Supplementary Material



## Notes

SEM images of BC and GC clay samples at different heat treatment temperature; EDS spectra of BC and GC clay samples; histograms of the particle size distribution of BC and GC samples at different calcination temperatures; colorimetric parameters of brown and gray clays calcined at different temperatures and applied as pigment (10%) in colorless paint and in white paint (PDF)
