# Supplementary material for: Compositional Properties and Colorimetric Characterization of Calcined Clays from the Central-West Region of Paraná and Their Application as Sustainable Pigment in Paints
Source: ACS Omega. 2025 Nov 3;10(44):53184–93. doi: 10.1021/acsomega.5c07843 (PMC12613131; doi:10.1021/acsomega.5c07843)
Supplement: Supplementary file 1 [file ao5c07843_si_001.pdf]

Supporting Information for "Compositional Properties and Colorimetric Characterization of Calcined Clays from the Central-West Region of Paraná and their Application as Sustainable Pigment in Paints"

Anne Raquel Sotiles[1*], Patrícia Appelt[1], Ricardo Schneider[2], Fauze Jacó Anaissi[1], Rafael Marangoni[1*]

[1]Department of Chemistry, Midwestern State University (UNICENTRO), Campus CEDETEG, Alameda Élio Antonio Dalla Vecchia, 85040-167, Guarapuava - PR, Brazil. E-mail: A.R.S.: anne.sotiles@gmail.com; P.A.: patriciaappelt@unicentro.br; F.J.A.: anaissi@unicentro.br; R.M.: rmarangoni@unicentro.br.

[2]Department of Chemistry, Federal Technological University of Paraná (UTFPR), Cristo Rei, 19, 85902-490, Toledo-PR, Brazil. E-mail: rikardos17@gmail.com.

Figure S1. SEM images of BC clay at different heat treatment temperatures, BC100 (A), BC200 (B), BC400 (C), BC600 (D), BC800 (E) and BC1000 (F).

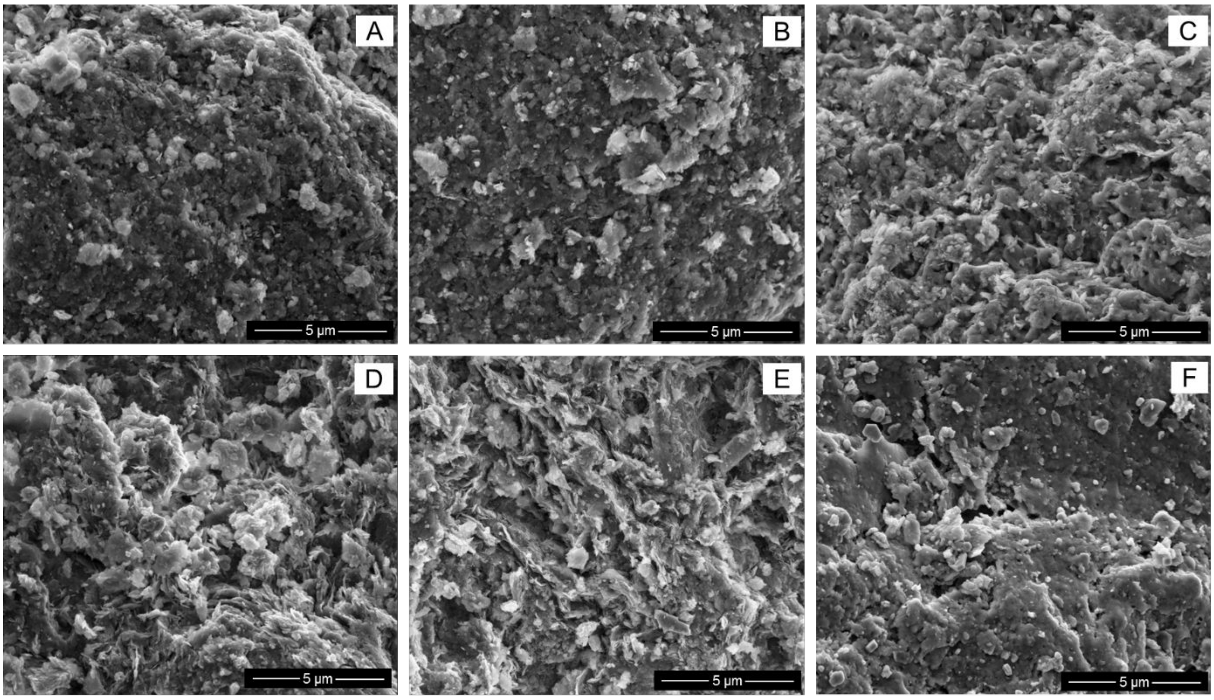

Figure S2. SEM images of GC clay at different heat treatment temperatures, GC100 (A), GC200 (B), GC400 (C), GC600 (D), GC800 (E) and GC1000 (F).

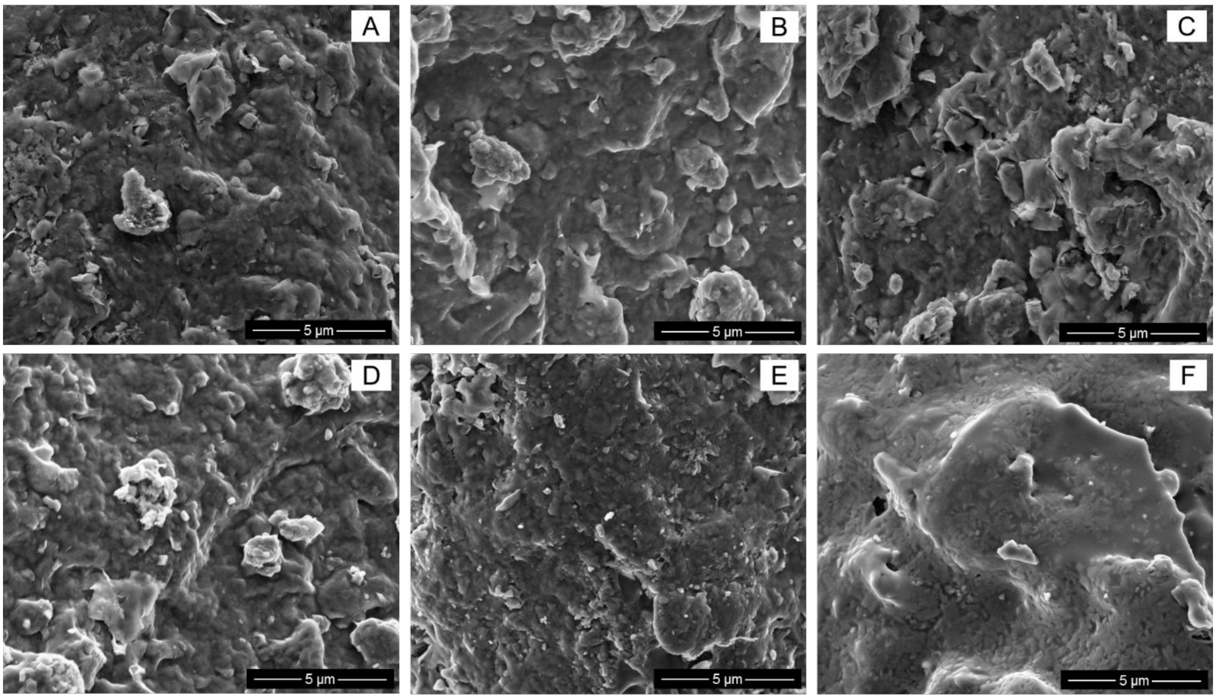

Figure S3. EDS spectra of BC (A) and GC (B) clay samples.

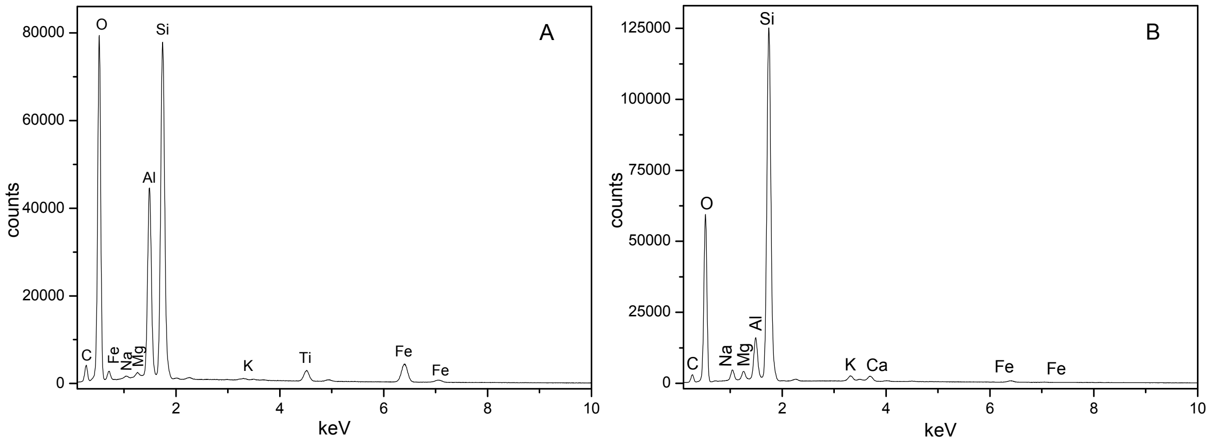

Figure S4. Histograms of the particle size distribution of BC samples at different calcination temperatures, BC100 (A), BC200 (B), BC400 (C), BC600 (D), BC800 (E) and BC1000 (F).

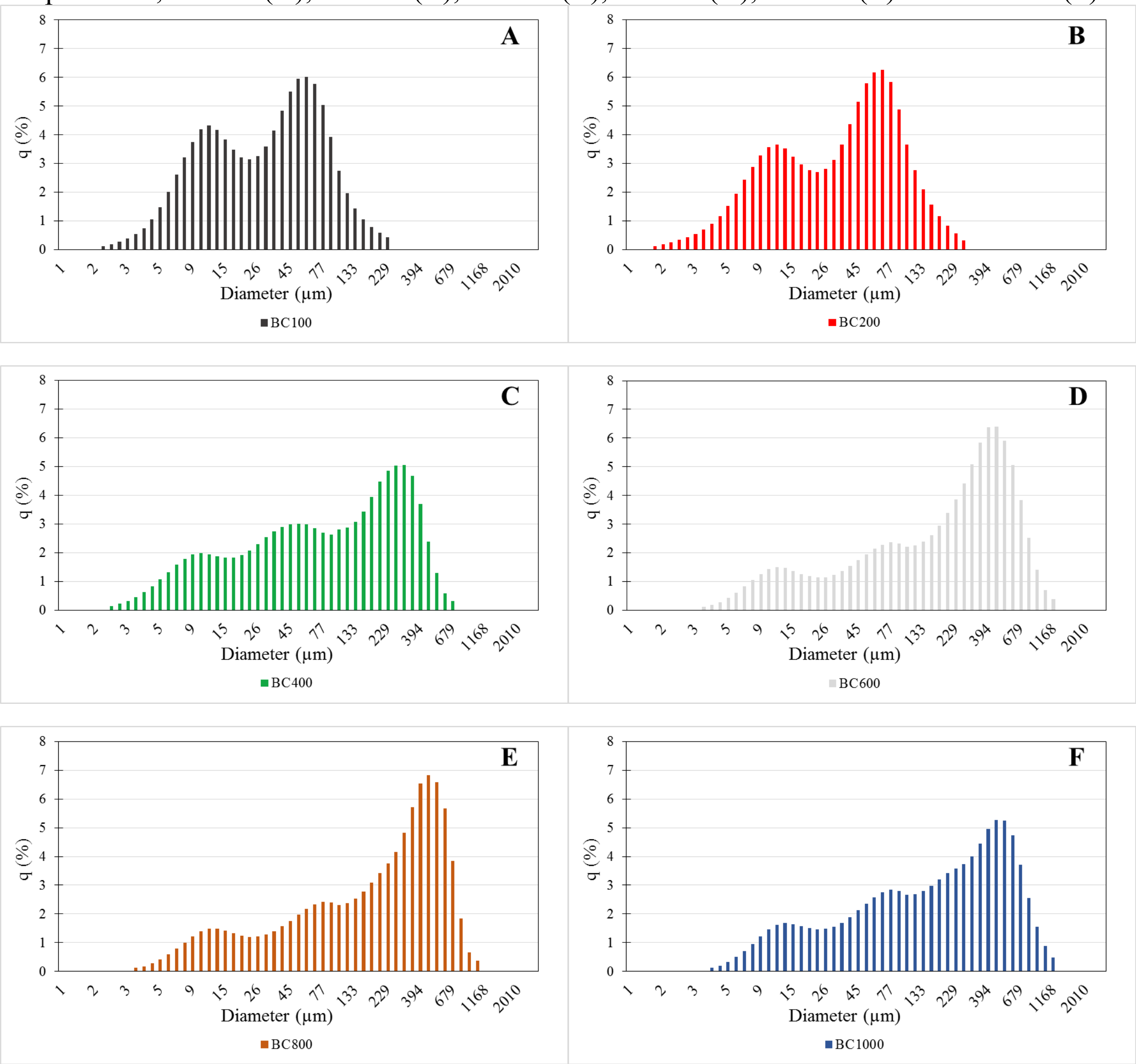

Figure S5. Histograms of the particle size distribution of GC samples at different calcination temperatures, GC100 (A), GC200 (B), GC400 (C), GC600 (D), GC800 (E) and GC1000 (F).

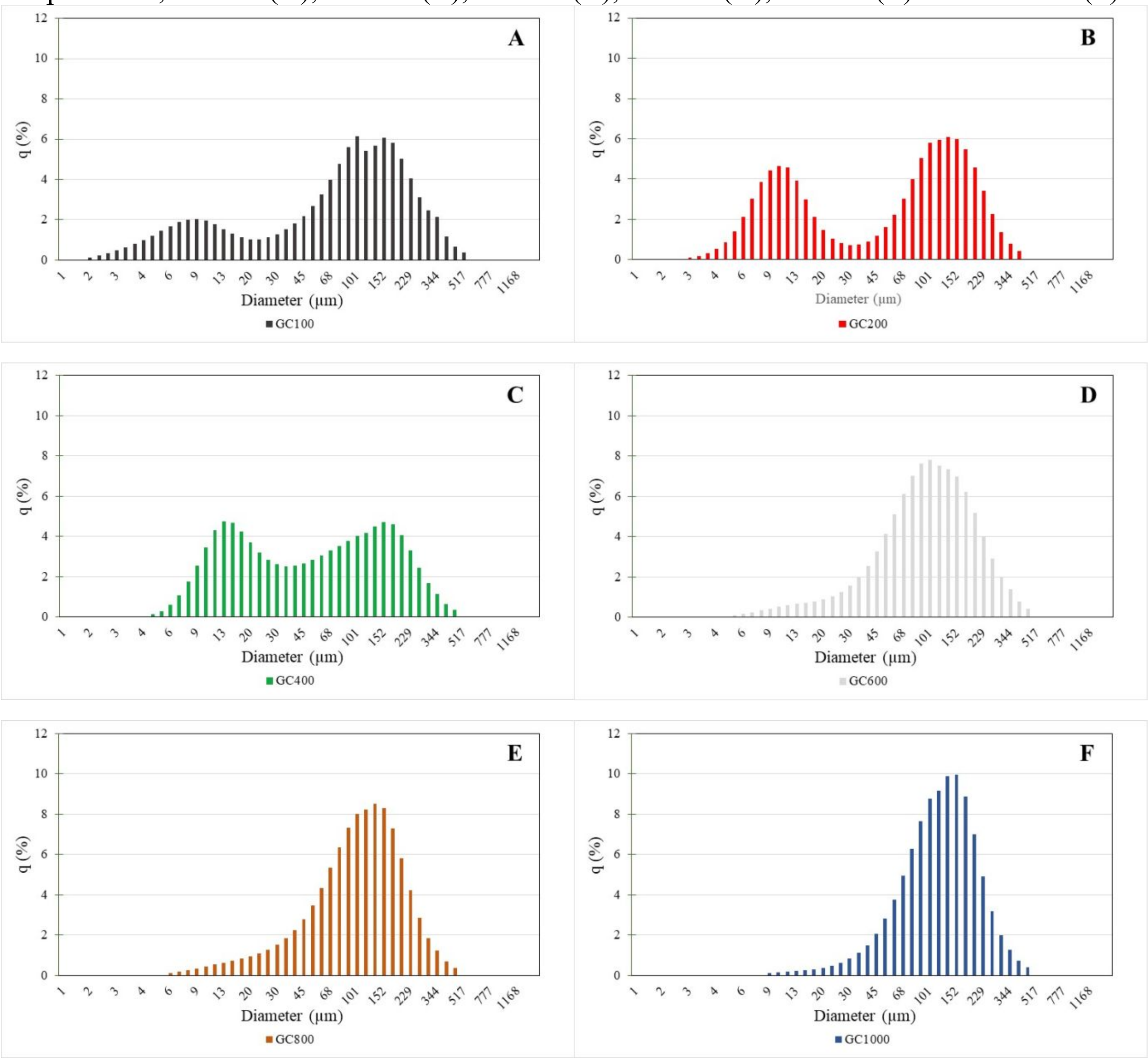

Table S1- Colorimetric parameters of brown and gray clays calcined at different temperatures and applied as pigment (10%) in colorless paint.

Sample	L	a*	b*	c*	h	ΔE	Color RGB
Colorless paint	85.56	0.35	5.05	5.06	86.04	-	
CPBC100	56.34	14.9	34.11	37.22	66.41	43.70	
CPBC200	50.28	15.54	30.02	33.81	62.63	45.81	
CPBC400	40.91	19.79	22.3	29.82	48.42	51.66	
CPBC600	69.24	17.43	29.4	34.17	59.34	33.93	
CPBC800	68.09	21.47	33.89	40.11	57.64	39.79	
CPBC1000	71.87	16.93	26.49	31.44	57.42	30.36	
CPGC100	81.17	0.17	7.18	7.18	88.67	4.88	
CPGC200	86.04	0.45	7.48	7.50	86.56	2.48	
CPGC400	79.16	3.06	14.27	14.59	77.91	11.55	
CPGC600	84.7	3.38	13.59	14.01	76.05	9.10	
CPGC800	83.51	2.88	11.08	11.44	75.45	6.85	
CPGC1000	83.76	4.42	13.13	13.86	71.38	9.22	

Table S2- Colorimetric parameters of brown and gray clays calcined at different temperatures and applied as pigment (10%) in white paint.

Sample	L	a*	b*	c*	h	ΔE	Color RGB
White paint	90.64	-0.76	2.44	2.56	107.17	-	
WPBC100	74.58	3.09	12.35	12.73	75.98	19.26	
WPBC200	73.99	3.42	12.51	12.97	74.69	19.90	
WPBC400	72.09	8.07	14.66	16.73	61.16	23.90	
WPBC600	79.99	6.94	10.78	12.82	57.23	15.56	
WPBC800	82.56	6.67	10.3	12.27	57.08	13.50	
WPBC1000	84.95	4.19	5.8	7.16	54.18	8.26	
WPGC100	89.26	-0.62	3.37	3.43	100.46	1.67	
WPGC200	88.23	-0.48	3.64	3.67	97.48	2.71	
WPGC400	85.64	0.38	4.79	4.81	85.48	5.64	
WPGC600	89.09	0.69	4.87	4.92	81.91	3.23	
WPGC800	86.4	1.02	4.63	4.74	77.56	5.09	
WPGC1000	89.49	0.84	4.47	4.55	79.35	2.83	
